# Diagnostic Performance of Seven Commercial COVID-19 Serology Tests Available in South America

**DOI:** 10.3389/fcimb.2022.787987

**Published:** 2022-02-18

**Authors:** Ismar A. Rivera-Olivero, Aquiles R. Henríquez-Trujillo, Nikolaos C. Kyriakidis, Esteban Ortiz-Prado, Juan Carlos Laglaguano, Alexander Paolo Vallejo-Janeta, Tannya Lozada, Miguel Angel Garcia-Bereguiain, UDLA COVID-19 team

**Affiliations:** ^1^ One Health Research Group, Universidad de Las Américas, Quito, Ecuador; ^2^ Decanato de Investigación y Vinculación, Universidad de las Américas, Quito, Ecuador

**Keywords:** SARS-CoV-2, COVID-19, serological test, ELISA, rapid test, diagnosis, Ecuador

## Abstract

**Background:**

Although RT-qPCR remains the gold-standard for COVID-19 diagnosis, anti-SARS-CoV-2 serology-based assays have been widely used during 2020 as an alternative for individual and mass testing, and are currently used for seroprevalence studies.

**Objective:**

To study the clinical performance of seven commercial serological tests for COVID-19 diagnosis available in South America.

**Methods:**

We conducted a blind evaluation of five lateral-flow immunoassays (LFIA) and two enzyme-linked immunosorbent assays (ELISAs) for detecting anti-SARS-CoV-2 antibodies.

**Results:**

We found no statistically significant differences among ELISA kits and LFIAs for anti-SARS-CoV-2 IgG sensitivity (values ranging from 76.4% to 83.5%) and specificity (100% for the seven serological assays). For anti-SARS-CoV-2 IgM, the five LFIAs have a significantly higher sensitivity for samples collected 15 days after the first time RT-qPCR positive test, with values ranging from 47.1% to 88.2%; moreover, the specificity varied from 85% to 100%, but the only LFIA brand with a 100% specificity had the lowest sensitivity.

**Conclusion:**

The diagnostic performance of the seven serological tests was acceptable for the seven brands tested for anti-SARS-CoV-2 IgG detection for seroprevalence screening purposes. On the other hand, our results show the lack of accuracy of anti-SARS-CoV-2 IgM detection in LFIAs as a tool for SARS-CoV-2 acute-phase infection diagnosis.

## Introduction

The detection of the novel coronavirus SARS-CoV-2 in the Chinese province of Hubei in December 2019 led to the Coronavirus Disease 2019 (COVID-19) outbreak that resulted in the World Health Organization (WHO) declaring a pandemic in March 11^th^ 2020 (Gorbalenya et al., 2020; Zhou et al., 2020). By the end of September 2021, more than 230 million cases and 4.7 million deaths have been reported worldwide (https://coronavirus.jhu.edu/map.html). The Americas is one of the most affected regions with millions of reported cases and deaths, and considering only the numbers for USA and Brazil, more than 63 million cases and 1.2 million of deaths have been reported (https://coronavirus.jhu.edu/map.html). In Ecuador, more than 500,000 cases and 32,000 deaths were reported by the end of September 2021 (https://www.salud.gob.ec/actualizacion-de-casos-de-coronavirus-en-ecuador/).

The insufficient SARS-CoV-2 testing capacity even at high-income countries during the first months of the COVID-19 pandemic has been suggested as one of the reasons for the dramatic scenario created by COVID-19 pandemic (Pullano et al., 2021). Control and prevention of SARS-CoV-2 transmission are the aims of any containment strategy, based in a testing and tracking approach as recommended by the World Health Organization. However, current numbers of cases and deaths related to the COVID-19 pandemic worldwide would suggest that these control and prevention strategies have been hampered by a lack of massive testing in several regions of world, particularly at low- and middle-income countries (Torres and Sacoto, 2020; Henriquez-Trujillo et al., 2021; Pullano et al., 2021). During the first semester of COVID-19 pandemic, SARS-CoV-2 genomic material detection by RT-qPCR was the main gold standard method available for COVID-19 diagnosis worldwide. This technique has significant logistic and capacity limitations like the need for sophisticated and expensive equipment, such as real time thermal cyclers, trained personnel, or permanent supply of expensive reagents. Thus, RT-qPCR-based SARS-CoV-2 testing capacity was limited even in high income countries during the first wave of the COVID-19 pandemic (Pullano et al., 2021). However, the Emergency Use Authorization of SARS-CoV-2 antigen tests since the end of 2020 and the worldwide improved capacity of reagents supply and RT-qPCR testing have partially overcome this problem.

These point-of-care rapid antigen tests became increasingly available, endorsed by regulatory agencies such as the Federal Drug Administration of the USA and have successfully replaced IgM serological testing as a rapid diagnostic tool for active SARS-CoV-2 infection detection (Cubas-Atienzar et al., 2021). However, serology is still a useful tool for epidemiological studies to determine the prevalence of infection in the general population or for screening of individuals who had a contact with SARS-CoV-2 infected people, but did not receive a confirmatory molecular test, to assist on vaccination policies (Watson et al., 2020).

In this context, numerous anti-SARS-CoV-2 serology-based assays, based on detection of antibodies and including point-of-care rapid diagnostic tests or conventional platforms, have recently become available and approved for clinical use worldwide, aiming to provide information about the individual seroprotection status but with a reduced sensitivity and specificity (WHO). These tests that detect anti-SARS-CoV-2 antibodies are typically based on lateral-flow immunoassays (LFIA) or enzyme-linked immunosorbent assays (ELISA). As an additional advantage, serological tests require less technical expertise and equipment, and have a much lower cost-per-patient diagnosis than RT-qPCR assays. Additionally, since the sample to be processed is whole blood collected in tubes or taken from fingerstick, they present a lower risk to the healthcare staff than collecting potentially infectious respiratory specimens for RT-qPCR. These advantages made serological tests widely used during 2020 even at middle- and low-income countries not only to detect previous infection (IgG seropositivity), but usually as a rapid diagnostic tool for ongoing SARS-CoV-2 infection (IgM seropositivity). However, the main disadvantage of serological test is related to lack of specificity due to cross reactivity with other pathogens, particularly for IgM detection, so the serodiagnostic power of antibodies against SARS-CoV-2 remains a topic of further research (Cota et al., 2020; Hou et al., 2020; Zhao et al., 2020).

Although clinical performance studies for COVID-19 diagnostic tests have become increasingly available, reports related to COVID-19 tests commercially available at low- or middle-income countries are still scarce (Cota et al., 2020; Deeks et al., 2020; Lisboa Bastos et al., 2020; Freire-Paspuel and Garcia-Bereguiain, 2021a; Freire-Paspuel and Garcia-Bereguiain, 2021b). In top of that, the high percentage of false-positive results of these tests, compromising their specificity, has been described for middle and low-income countries associated to higher prevalence of certain infectious diseases (Echeverría et al., 2021; Tso et al., 2021), and also at tropical latitudes associated to endemic infections caused by arboviruses (Faccini-Martínez et al., 2020). For these reasons, locally assessed clinical performance studies are necessary, especially for regions like South America where there is a single study of this kind to the best of our knowledge (Cota et al., 2020). The aim of this work was to evaluate the clinical performance of seven COVID-19 serology available in South American countries including Ecuador.

## Methods

### Study Design

In the present panel-based study, two panels of specimens were used. A “COVID-19 positive panel” formed by 127 serum samples collected 15 and/or 30 days following positive SARS-CoV-2 detection by RT-qPCR, performed at the diagnostic laboratory of “Universidad de Las Américas”, as previously reported (Freire-Paspuel and Garcia-Bereguiain, 2021a; Freire-Paspuel and Garcia-Bereguiain, 2021b; Freire-Paspuel et al., 2021; Freire-Paspuel B and Garcia-Bereguiain MA, 2021). A “COVID-19 negative panel” including 40 sera samples collected in the pre-pandemic period prior to June 2019. This samples were randomly selected from a sera bank from asymptomatic individuals included in previous seroprevalence studies. Only one sample per individual was included in each of the panels, and all the samples included in the study were from individuals living in Ecuador.

### Serological Assays

Two groups of serological assays were included in this study:

- Lateral Flow Immunossays (LFIAs). Five commercially available LFIAs for SARS-CoV-2 IgM/IgG detection were evaluated. At the time of testing, for each tested IgM/IgG one cartridge per sample were labeled by a randomized sample number. The appropriate sample volume was transferred from the tube to the indicated sample port, followed immediately by provided diluent, following manufacturer´s instructions. The lateral flow cartridges were incubated for the recommended time at room temperature before readings. Cartridges were read for test line intensity by two independent readers blinded to specimen status, according to manufacturer´s instructions. Briefly, the tests tested, volumes of sample, and the time to read the results were as follows: LFIA 1: for Artron Laboratories Inc. (Burnaby, British Columbia, Canada), 10 ul of serum sample were applied on sample well and IgG/IgM responses were read after 15-20 minutes, but no later than 30 min. LFIA 2: for Biohit Healthcare Co.Ltd (Hefei, Anhui Province, China), 10 ul of serum sample were added in the sample hole and results were read within 15 minutes. LFIA 3: for Camtech Diagnostics Pte Ltd (Henderson, Singapore), 10 ul of serum sample were added in each sample well (1 sample well for IgG/1 sample well for IgM, 2 sample well per cassette) and results were read after 10 minutes, but no later than 18 minutes. LFIA 4: for INNOVITA (TANGSHAN) Biological Technology Co.,Ltd (Hebei, China), 10 ul of serum sample were added on each sample well (1 sample well for IgG/1 sample well for IgM, 2 sample well per cassette) and results were read within 15 minutes. LFIA 5: for Zybio Inc (Dadukou District, Chongquing, China), 5 ul of serum sample were added to the sample well and results were read within 15 minutes.

- ELISA Tests. Two different commercially available ELISA kits were included in the study. ELISA Kit 1: COVID-19 IgG Enzyme InmunoAssay manufactured by Dia Pro Diagnostic Bioprobes S.r.l. (Sesto San Giovanni, Milan, Italy) for the determination of IgG antibodies against the SARS-CoV-2-specific nucleocapsid (core) and spike antigens. The test was performed as per manufacturer’s instructions. The internal controls (Negative control, Positive control, and blank well) were tested every time the kit was used to verify whether their OD values matched the manufacturer´s requirements. If OD values were within the expected range, the test results were calculated by means of a cut-off value; after that test results were interpreted as a ratio of sample OD/Cut-off OD. A positive result was assigned to ratios >1.1. A negative result is assigned to ratio values < 0.9. An undetermined result was assigned to ratio values within the range 0.9-1.1. ELISA Kit 2: ID Screen^®^. SARS-CoV-2 -N IgG Indirect manufactured by IDVet (Grabels, France) for the specific detection of IgG antibodies against the nucleocapsid of SARS-CoV-2. The test was performed as per manufacturer’s instructions. A ratio sample OD (S)/positive control OD (P) was calculated for each sample. The results are analyzed as follows: positive S/P ≥ 40%; negative S/P % ≤ 30% Negative; undetermined 30% < S/P < 40%.

### Statistical Analysis

IC intervals for 95% probability values for sensitivity and specificity were calculated individually for the sensitivity and specificity values using Jamovi software.

## Results

An evaluation of the clinical performance of 7 commercial serological test for COVID-19 diagnosis was carried out using 167 sera, including 127 sera from SARS-CoV-2 RT-qPCR positive individuals (positive panel) and 40 sera sampled before 2020 (negative panel). For the positive panel, a stratification of the results was carried out in terms of the time between first RT-qPCR positive result and sera sampling. Two groups were defined at 15 days and 30 or more days post-detection of SARS-CoV-2 infection. **Tables 1** and **2** summarize the performance of 7 serological kits tested, including 2 ELISA kits for the detection of anti-SARS-CoV-2 IgG and five LFIAs for anti-SARS-CoV-2 IgM and IgG detection.

**Table 1:** Diagnostic performance of SARS-CoV-2 IgG/IgM lateral flow immunoassays.

Performance parameter	Brand				
	Artron™	BioHit™	Camtech™	Innovita™	Zybio™
**IgG antibodies detection**					
Overall sensitivity - % (IC95%)	76.4 (68 - 83.5)	76.4 (68 - 83.5)	80.2 (72.1 - 86.7)	79.5 (71.5 - 86.2)	80.3 (72.3 - 86.8)
*Sensitivity at 15 days*	76.5 (58.8 - 89.3)	82.4 (65.5 - 93.2)	79.4 (62.1 - 91.3)	85.3 (68.9 - 95.1)	50.0 (32.4 - 67.6)
*Sensitivity post-infection at 30 days*	76.3 (66.4 - 84.5)	74.2 (64.1 - 82.7)	79.6 (70 - 87.2)	77.4 (67.6 - 85.5)	72.3 (62.2 - 81.1)
Overall specificity - % (IC95%)	100.0 (84.6 - 100.0)	100.0 (91.2 - 100.0)	100.0 (91.2 - 100.0)	100.0 (91.2 - 100.0)	100.0 (91.2 - 100.0)
**IgM antibodies detection**					
Overall sensitivity - % (IC95%)	59.8 (50.8 - 68.4)	63.8 (54.8 - 72.1)	46.8 (37.9 - 55.9)	79.5 (71.5 - 86.2)	40.9 (32.3 - 50)
*Sensitivity at 15 days*	70.6 (52.5 - 84.9)	88.2 (72.6 - 96.7)	67.6 (49.5 - 82.6)	76.5 (58.8 - 89.3)	47.1 (29.8 - 64.9)
*Sensitivity post-infection at 30 days*	55.9 (45.2 - 66.2)	54.8 (44.2 - 65.2)	38.7 (28.8 - 49.4)	38.7 (28.8 - 49.4)	25.8 (17.3 - 35.9)
Overall specificity - % (IC95%)	95.5 (77.2 - 99.9)	85.0 (70.2 - 94.3)	97.5 (86.8 - 99.9)	92.5 (79.6 - 98.4)	100.0 (91.2 - 100.0)

**Table 2:** Diagnostic performance of SARS-CoV-2 IgG ELISA tests.

Performance parameter	Brand	
	DiaPro™	IDVet™
Overall sensitivity - % (IC95%)	82.7 (74.9 - 88.8)	83.5 (75.8 - 89.5)
*sensitivity at 15 days*	82.4 (65.5 - 93.23)	88.2 (72.6 - 96.7)
*sensitivity at 30 days*	83.9 (74.8 - 90.7)	82.8 (73.6 - 89.8)
Overall specificity - % (IC95%)	100.0 (91.2 - 100.0)	100.0 (91.2 - 100.0)

### Clinical Performance of Lateral Flow Immunoassays (LFIAs)

The results of the evaluation of the five LFIAs are detailed in **Table 1**. For anti-SARS-Cov-2 IgG detection, the overall specificity of the five brands was 100%, while the overall sensitivity ranged from 76.4% (68-85.5 IC 95%) to 80.3% (72.3-86.8 IC 95%), although no statistically significant differences were found among the five LFIAs brands. Four of the five LFIAs brands did not show statistically significant differences for the sensitivity values for SARS-CoV-2 positive samples between 15 days samples or 30 or more days samples since RT-qPCR positivity. However, for the “Zybio” brand, there was a significant increase (p<0.05) in sensitivity from 50% (32.4-67.6 IC 95%) at 15 days to 72.3% (62.2-81.8 IC95%) at 30 or more days after a RT-qPCR positive result.

For anti-SARS-CoV-2 IgM detection, the overall specificity of four of the five LFIAs brands was over 92.5%, while this value for “Zybio” brand was 85.5% (70.2-94.3 IC95%), although those differences were not statistically significant. The sensitivity for positive samples collected 15 days after the RT-qPCR test ranged from 47.1% (29.8-64.9 IC95%) to 88.2% (72.6-96.7 IC95%). Furthermore, there was a statistically significant (p<0.05) reduction in the sensitivity values for the five LFIAs brands for samples collected 30 or more days after the RT-qPCR positive result.

### Clinical Performance of ELISA Tests

The results of the evaluation of the different tests are detailed in **Table 2**. For anti-SARS-Cov-2 IgG detection, the overall sensitivity of the two brands was neither statistically significant between them nor compared to LFIAs. For both ELISA kits, there were no statistically significant differences of the sensitivity values among samples collected 15 days or 30 or more days after the RT-qPCR test. The overall sensitivity was 82.7% (74.9-88.8 IC 95%) and 83.5% (75.8-89.5 IC 95%) for “DiaPro” and “IDVet” brands, respectively. Moreover, both ELISA kits had a specificity of 100%.

**Figure 1:**
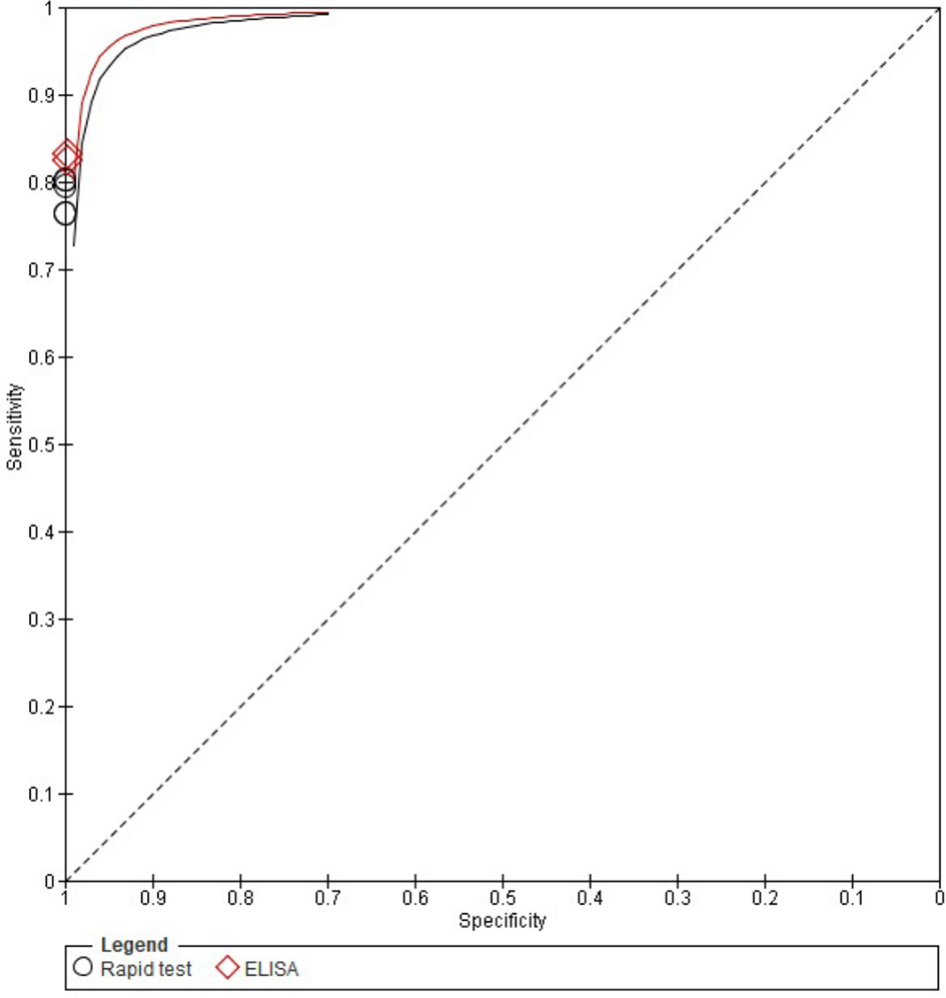
ROC curves for anti-SARS-CoV-2 IgG detection of the seven commercial COVID-19 serology test available included in this study. ELISA kits are shown in red. LFIAs are shown in black.

**Figure 1** includes the ROC curves for the five LFIAs and two ELISA kits tested for IgG detection, showing that the ELISA kits had a slightly higher sensitivity, although it was not found to be statistically significant.

## Discussion

To our knowledge, this is the first report addressing the clinical performance of serological tests for COVID-19 diagnosis commercially available in Ecuador and other South American countries like Colombia and Peru. Although there are some reviews already published on the subject (Cota et al., 2020; Deeks et al., 2020), there is only one similar study carried out in South America, specifically in Brazil, including a different set of serological test brands (Lisboa Bastos et al., 2020). Local accuracy data based on real scenarios are essential given the marked regional differences reported for the performance of the tests. This issue is specially relevant in tropical regions and/or middle- and low-income countries where a higher prevalence of certain infectious diseases is expected, potentially compromising the specificity of the serological tests (Cota et al., 2020; Faccini-Martínez et al., 2020; Echeverría et al., 2021; Tso et al., 2021). For instance, lack of specificity due to cross reactivity with Zika and Dengue positive sera samples have been described, ranging form 2% to 26% for IgG and IgM depending on the commercial brands (Cota et al., 2020; Faccini-Martínez et al., 2020). Additionally, local clinical evaluations are also required for COVID-19 related tests in South America, since several RT-qPCR kits and serological tests either did not receive or had their clinical use authorization revoked at their countries of production (Cota et al., 2020; Freire-Paspuel and Garcia-Bereguiain, 2021a; Freire-Paspuel and Garcia-Bereguiain, 2021b).

In our study, we did not report a lack of specificity for the seven serological tests analyzed for anti-SARS-CoV-2 IgG detection. However, for anti-SARS-CoV-2 IgM detection, the specificity was lower than 100% for four of the five LFIA brands evaluated. Moreover, only one of the brands evaluated maintained a 100% specificity for IgM detection, although in that case the reduction in sensitivity for IgM detection was over 50%. On the other hand, although we did not find statistically significant differences among ELISA and LFIAs kits for anti-SARS-CoV-2 IgG sensitivity, the values obtained (ranging from 76.4% to 83.5%) were clearly below the high sensitivity values (over 90%) reported by manufacturers. However, the sensitivity values for the serological tests included in this study, are higher than the values reported for some serological kits used in Brazil (Cota et al., 2020). The sensitivity values were lower for anti-SARS-CoV-2 IgM detection, with only two LFIAs presenting a sensitivity ≥75% even for samples collected 15 days after a positive RT-qPCR test. However, these brands were found to have a strong reduction of specificity, with values of 92.5 and 85%, respectively.

Overall, the clinical performance of ELISA kits and LFIAs was quite similar, with a slight increase in sensitivity for anti-SARS-CoV-2 IgG detection by ELISA. So far, regarding the choice between ELISA kits or LFIAs, logistical issues and cost evaluation should be considered. For instance, although this study did not evaluate the direct point-of-care use with finger peripheral blood for LFIAs, this is something recommended by the manufacturers. If the sensitivity of LFIAs is maintained for this alternative type of use, their cost-effectiveness would definitely compensate their lower sensitivity compared to the ELISA kits.

Regarding the potential use of these serological tests in the current scenario of availability of highly specific and cheap SARS-CoV-2 antigen test (Cubas-Atienzar et al., 2021), our results clearly endorse the inadequacy of the use of anti-SARS-CoV-2 IgM antibodies as markers of active SARS-CoV-2 infection, as it has also been suggested by other reports (Cota et al., 2020). On the other hand, the high specificity and the acceptable sensitivity values obtained for anti-SARS-CoV-2 IgG, considering that antibodies release is not the only immune response to COVID-19 infection (GeurtsvanKessel et al., 2020; Turner et al., 2021), suggest that the serological COVID-19 tests included in our study can be useful tools for seroprevalence studies. Estimating the percentage of the population that has already been infected in the community is essential for understanding the spread of the pandemic, and will also assist vaccination program decisions in middle- and low-income countries (Santander-Gordon et al., 2021).

In conclusion, our results reveal no significant differences in terms of sensitivity and specificity for anti-SARS-CoV-2 IgG detection among ELISA kits and LFIAs. The overall clinical performance obtained for the seven serological tests included in the study was worse than promised by manufacturers. However, with an overall specificity of 100% and sensitivity values over 75% for anti-SARS-CoV-2 IgG detection, these tests are an affordable and useful tool for seroprevalence studies in the context of middle- and low-income countries like Ecuador.

## UDLA COVID-19 Team

Byron Freire-Paspuel, Barbara Coronel, Heberson Galvis, Tatiana Jaramillo, Maria Belen Rodriguez Paredes, Angel S. Rodriguez Pazmiño, Diana Morales-Jadan, Daniela Santander Gordon, Gabriel Alfredo Iturralde, Julio Alejandro Teran, Karen Marcela Vasquez, Jonathan Dario Rondal, Genoveva Granda, Ana Cecilia Santamaria, Cynthia Lorena Pino, Oscar Lenin Espinosa, Angie Buitron, David Sanchez Grisales, Karina Beatriz Jimenez, Vanessa Bastidas, Dayana Marcela Aguilar, Ines Maria Paredes, Christian David Bilvao, Henry Herrera, Pablo Marcelo Espinosa, Edison Andres Galarraga, Marlon Steven Zambrano-Mila, Ana Maria Tito, Nelson David Zapata.

## Data Availability Statement

The original contributions presented in the study are included in the article/supplementary material. Further inquiries can be directed to the corresponding author.

## Ethics Statement

This study was approved by the IRB from the Dirección Nacional de Inteligencia de la Salud (Ministerio de Salud Publica, Ecuador) under the code 008-2020. The patients/participants provided their written informed consent to participate in this study.

## Author Contributions

IR-O and MG-B wrote the manuscript. All the authors have contributed to the experimental design, data collection and analysis, and also reviewing the final version of the manuscript. All authors contributed to the article and approved the submitted version.

## Funding

This study was funded by Universidad de Las Américas and by Fundación CRISFE (Fondo “Sumar juntos”).

## Conflict of Interest

The authors declare that the research was conducted in the absence of any commercial or financial relationships that could be construed as a potential conflict of interest.

## Publisher’s Note

All claims expressed in this article are solely those of the authors and do not necessarily represent those of their affiliated organizations, or those of the publisher, the editors and the reviewers. Any product that may be evaluated in this article, or claim that may be made by its manufacturer, is not guaranteed or endorsed by the publisher.
